# Promoting Reading Achievement in Children With Developmental Language Disorders: What Can We Learn From Research on Specific Language Impairment and Dyslexia?

**DOI:** 10.1044/2020_JSLHR-20-00118

**Published:** 2020-10-15

**Authors:** Suzanne M. Adlof

**Affiliations:** a University of South Carolina, Columbia

## Abstract

**Purpose:**

Specific language impairment (SLI; see also developmental language disorder) and dyslexia are separate, yet frequently co-occurring disorders that confer risks to reading comprehension and academic achievement. Until recently, most studies of one disorder had little consideration of the other, and each disorder was addressed by different practitioners. However, understanding how the two disorders relate to each other is important for advancing theories about each disorder and improving reading comprehension and academic achievement. The purpose of this clinical focus article is to integrate research on SLI and dyslexia as well as advocate for the consideration of comorbidities in future research and clinical practice.

**Method:**

The first section reviews definitions as well as inclusionary and exclusionary criteria for SLI and dyslexia. The second section reviews research demonstrating that SLI and dyslexia are different disorders that often co-occur. Studies examining language, working memory, and academic achievement in children with separate versus co-occurring SLI and dyslexia are reviewed. The final section compares and contrasts school identification frameworks for children with SLI and dyslexia and considers the potential benefits of incorporating broad language skills into response to intervention (RTI) assessment frameworks.

**Conclusions:**

Children with weak language skills are at a high risk of experiencing reading problems, but language difficulties are often hidden from view. Directly addressing language skills within school RTI frameworks can help improve the identification and treatment of children with SLI and dyslexia as well as support improved reading comprehension and academic achievement for all students.

**Presentation Video:**

https://doi.org/10.23641/asha.13063793

Specific language impairment (SLI) is defined by the National Institute on Deafness and Other Communication Disorders (2019) as “a language disorder that interferes with the development of language skills in children who have no hearing loss or intellectual disabilities.” The estimated prevalence of SLI in kindergarten students in the United States is 7.4% (Tomblin et al., 1997). Children with SLI comprise the majority of children with developmental language disorder (DLD^1^; Norbury et al., 2016) and are at significantly increased risk of having reading disabilities (Catts et al., 2002; Snowling et al., 2000) as well as experiencing difficulties in other academic areas including writing, math, and science (Cross et al., 2019; Dockrell et al., 2011; Durkin et al., 2015; G. J. Williams et al., 2013). In combination, these difficulties place affected children at risk for lower educational attainment and restricted employment opportunities, compared to their peers with typical language abilities (Conti-Ramsden & Durkin, 2012; Johnson et al., 2010).

The majority of children with SLI struggle with reading comprehension at some point in development, but there is heterogeneity in the proximal causes of their reading comprehension difficulties (Catts et al., 2012; Kelso et al., 2007). This heterogeneity is predicted by the “simple view of reading” (Gough & Tunmer, 1986; Hoover & Gough, 1990), which states that reading comprehension is the product of two component skills, namely, “decoding” (the ability to translate strings of printed letters into pronounceable words) and “linguistic comprehension,” which is defined by Gough and Tunmer (1986) as “the process by which, given lexical (i.e., word) information, sentences and discourses are interpreted” (p. 7). This latter component is often referred to as “listening comprehension” because the authors suggested that listening comprehension tasks could be used to measure the understanding of a text when it is heard instead of read (Gough & Tunmer, 1986). Both decoding and listening comprehension are necessary for reading comprehension across development, but their relative importance shifts as the texts children encounter increase in complexity and as the topics they read about become less familiar (Adlof et al., 2006; Foorman et al., 2018; Language and Reading Research Consortium, 2015). In the primary grades, when children are “learning to read,” texts are usually constructed with language simpler than what children are able to comprehend in the spoken domain. Thus, reading comprehension is primarily constrained by word reading. However, beginning around the second or third grade, as most children are able to read words accurately and fluently and as children are expected to “read to learn,” reading comprehension is primarily constrained by oral language skills.

Because they have a language disorder, most children with SLI are expected to struggle with listening comprehension. Some children with SLI also struggle with word reading, performing comparably to children with dyslexia (Catts et al., 2005; McArthur et al., 2000). However, many children with SLI perform quite well with word reading, demonstrating the ability to accurately and fluently decode both real words and pseudowords often at above-average levels (Bishop et al., 2009; Catts et al., 2005). For this latter group of children, reading comprehension difficulties may not be apparent until the later school grades, that is, when decoding is no longer the primary driver of reading comprehension (Catts et al., 2012). It is important to raise awareness of these different profiles (i.e., language impairment with good word reading vs. poor word reading) because they both need support of different kinds to develop proficiency in reading comprehension.

This clinical focus article compares and contrasts SLI and dyslexia, a disorder involving impaired word reading skills (Lyon et al., 2003; NICHD, 2020). SLI and dyslexia are different disorders that frequently co-occur (Catts et al., 2005). As predicted by the simple view of reading, both disorders have a negative impact on reading comprehension and confer risks to broader academic achievement. Until recently, most studies of one disorder had little to no consideration of the other, and each disorder was addressed by different practitioners. However, understanding how the two disorders relate to each other is important for advancing theories about each disorder and for improving instructional outcomes. The first section of this clinical focus article reviews definitions as well as inclusionary and exclusionary criteria for SLI and dyslexia. The second section reviews studies that have directly compared individuals with SLI and/or dyslexia. These studies demonstrate that SLI and dyslexia are different disorders that frequently co-occur, examine whether they are distinguishable based on other language or cognitive factors, and compare their functional academic outcomes. The final section considers how current school identification procedures differ for children with difficulties in oral language (e.g., SLI) versus word reading (e.g., dyslexia) and the implications of those procedures for theoretical and practical research.

## Defining SLI and Dyslexia

SLI and dyslexia are different disorders, but there are some similarities in their definitions. Both are defined based on the presence of a significant impairment in the skill of interest, that is, understanding and producing language in the case of SLI (Leonard, 2014; National Institute on Deafness and Other Communication Disorders, 2019) and accurate and fluent word reading and spelling in the case of dyslexia (Lyon et al., 2003; NICHD, 2020). Whereas there are many possible reasons why a child might struggle with spoken or written language, these two disorders have been described as “unexpected” (Lyon et al., 2003) or “unexplained” (Bishop, 2014) disorders because they occur without an obvious cause. That is, both disorders exclude factors such as hearing loss, acquired brain injury, or intellectual disabilities that might be expected to impact language or reading development (Bishop, 2006; Leonard, 2014). It is also presumed that children with SLI or dyslexia have been given adequate learning opportunities to acquire developmentally appropriate language and reading skills (Bishop, 2006; Leonard, 2014). However, the operationalization of these exclusionary criteria is challenging and has varied over time in studies of both disorders (Lopes et al., 2020; Reilly et al., 2014).

### Nonverbal Intelligence

Early case studies of children who could today be labeled as having dyslexia remarked on their significant difficulties in reading despite otherwise apparently average intelligence (W. P. Morgan, 1896; Orton, 1928). Such children could be viewed as exceptions to a common assumption that general intelligence set a ceiling on all aspects of cognitive development. Over time, dyslexia came to be diagnosed based on a discrepancy between IQ scores and reading achievement scores; because reading and verbal abilities were known to be correlated, the discrepancy was often considered on the basis of nonverbal IQ measures. However, there were multiple problems with discrepancy-based diagnostic approaches, including that they were unreliable, that there was little evidence of qualitative differences in foundational reading skills between those with and without a discrepancy, and that the presence or absence of a discrepancy did not predict a child's response to instruction (Francis et al., 2005; Stanovich, 1991; Stuebing et al., 2009). Similar themes can be seen in research related to childhood language disorders over the last 4 decades (Cole et al., 1990; Plante, 1998).

There is widespread agreement that nonverbal IQ should not determine who receives educational supports for language or reading difficulties, and the 2004 reauthorization of the Individuals with Disabilities Education Act (Public Law 108-446) removed the requirement of a discrepancy between IQ and achievement to qualify for public school special education services. To date, there has been little evidence that nonverbal IQ provides meaningful information about children's response to evidence-based interventions (Stuebing et al., 2009; note that much of this research has focused on interventions targeting word reading skills). Still, the relations between verbal and nonverbal cognitive skills remain of interest to researchers involved in the study of language, reading, and other learning disorders (Archibald et al., 2019; Durant et al., 2019; Justice et al., 2017; Korpipää et al., 2017; Peng et al., 2019). Studies of SLI and dyslexia frequently apply nonverbal IQ cutoffs as part of their exclusionary criteria (Gallinat & Spaulding, 2014; Lopes et al., 2020). These cutoffs have been particularly important for studies aimed at understanding causal mechanisms of language or reading impairment (Leonard, 2020; Rice, 2020). For example, a long history of studies employing this approach has identified important characteristics associated with dyslexia (e.g., poor phonemic awareness) and SLI (e.g., grammatical weaknesses). As explained by Bishop (2014), the significance of these characteristics might not have been appreciated had they only been observed in children whose poor reading or language performance was associated with low nonverbal IQ.

### Learning Opportunities

Diagnoses of SLI and dyslexia are made under the presumption that children have had adequate opportunities to learn. However, what constitutes an adequate learning opportunity differs between the two disorders. Oral language typically develops without formal instruction, provided that the child is exposed to verbal communication (Casillas et al., 2020; Shneidman et al., 2012), and proficiency is facilitated by meaningful communication exchanges with caregivers (Weisleder & Fernald, 2013). Children with normal hearing abilities are exposed to verbal communication from birth—and even in utero—and typically speak in complex, grammatically correct sentences by 4 years of age (Arndt & Schuele, 2013). Children with SLI can be found across socioeconomic strata, including highly educated households, and yet show difficulty acquiring age-appropriate grammar, vocabulary, and discourse skills (Norbury et al., 2016; Tomblin et al., 1997). However, most of what is known about SLI has been based on the study of monolingual, English-speaking children for whom much is known about the typical course of language development and for whom well-validated assessments of language abilities exist. Multilingual households are rapidly increasing in the United States, and the proportion of English learners in schools increased by 28% from 2001 to 2017 (National Clearinghouse for English Language Acquisition, 2020). The milestones of typical language development vary for children learning more than one language, and so, current challenges for the field are to fill this gap in the research and develop methods for identifying disordered language development in children learning more than one language (Muñoz et al., 2014).

Relative to spoken language, written language skills (i.e., reading and writing) develop years later. Literacy builds on the foundation of spoken language, and it is generally agreed among scientists and experts that learning to read in English requires explicit instruction (National Reading Panel, 2000; Rose, 2006). In English and other alphabetic writing systems, letters map onto phonemes. Some languages such as Spanish are described as having a shallow orthography because there is a near 1:1 correspondence between phonemes and the letters that represent them (i.e., graphemes). However, English is fairly opaque because many phonemes can be represented by more than one grapheme (e.g., /s/ can be spelled with “c” or “s”) and because many graphemes can represent more than one phoneme (e.g., “th” can be pronounced /θ/ or /ð/). Although a small minority of children may be able to learn these spoken–written language connections implicitly through exposure, most children learning to read English require explicit and systematic instruction in the relationships between phonemes and the letters that represent them. The scientific consensus on learning to read in English is that *explicit* and *systematic* instruction in the foundational skills that support fluent word reading should be universally provided to all students (Gersten et al., 2008; National Reading Panel, 2000; Rose, 2006). All children should be explicitly taught the relationships between phonemes and graphemes, and the progression of instruction should follow a logical sequence, that is, from the simplest and most frequently occurring forms to more complex and less frequently occurring forms. Despite this scientific consensus, many popular reading curricula do not include explicit and systematic instruction, making it challenging to differentiate individuals who have an innate difficulty learning to read from those who have had poor instruction (Castles et al., 2018).

## Research Comparing and Contrasting SLI and Dyslexia Subgroups

For the most part, SLI and dyslexia have been researched, identified, and treated separately. Numerous studies have been conducted to examine causal mechanisms and identify characteristics that could serve as clinical markers of each disorder. Whereas SLI affects multiple aspects of language at the word, sentence, and discourse levels, two clinical markers have received the most attention: grammatical difficulties, particularly with tense and agreement marking (Leonard, 2014; Rice et al., 2004), and nonword repetition difficulties (Estes et al., 2007). Dyslexia has been strongly linked to difficulties in the phonological component of language (Lyon et al., 2003; Snowling, 1998). These phonological difficulties have been described as core deficits, interfering with the mapping of phonemes to graphemes that is necessary for decoding new words and thereby playing a causal role in dyslexia (Vellutino et al., 2004).

Toward the end of the 20th century, researchers noted that children with SLI often showed poor word reading and that children who were later identified as having dyslexia often showed earlier weaknesses in vocabulary, grammar, and discourse skills. Such observations led researchers to question whether SLI and dyslexia were in fact distinct disorders or different manifestations of the same underlying deficit (Bishop & Snowling, 2004; Kamhi & Catts, 1986; McArthur et al., 2000). For example, McArthur et al. (2000) found that 55% of dyslexic children recruited from schools or clinics also met the criteria for SLI and that 51% of children with SLI recruited from language development centers also met the criteria for dyslexia. These authors hypothesized that whether a child was diagnosed with dyslexia or SLI depended more on who was giving the diagnosis than on specific characteristics of the child's performance.

Strong evidence that the two disorders are in fact separable was first provided in a large-scale, longitudinal study by Catts et al. (2005), which drew from a population-based sample of over 500 kindergarten students who were followed through eighth grade. The children were classified on the basis of a comprehensive language assessment in kindergarten and word reading evaluations in the second, fourth, and eighth grades. Results showed that 17%–36% of children with SLI in kindergarten developed dyslexia in a later grade and that 14%–19% of children with dyslexia in any of the later grades had SLI in kindergarten. Although these levels of overlap were significantly higher than chance, it was not the case that the two disorders were equivalent. Rather, there was wide variation in the language skills of children with dyslexia and in the word reading skills of children with SLI, such that some children with disorders in one domain had quite strong skills in the other domain. Interestingly, children with SLI who had good word reading skills (SLI-only) had similar levels of language impairment as children with combined SLI and dyslexia (SLI + dyslexia), but they were less likely to have received clinical services in the primary grades.

More recent studies have provided converging evidence that SLI and dyslexia can be distinguished on the basis of their defining characteristics, that is, impaired oral language or impaired word reading (Adlof et al., 2017; Baird et al., 2011; Bishop et al., 2009; De Groot et al., 2015; Eisenmajer et al., 2005; Fraser et al., 2010; McArthur & Castles, 2013; Ramus et al., 2013). An overarching theoretical question has been whether children with SLI and dyslexia can also be distinguished on the basis of some other underlying factor, such as phonological processing (Bishop & Snowling, 2004; Kamhi & Catts, 1986; Tallal et al., 1997). It is also of practical interest whether and how functional outcomes differ for individuals with SLI or dyslexia. This section reviews research that compares and contrasts language and cognitive skills as well as academic achievement in subgroups of children with separate or co-occurring SLI and dyslexia. Following the publication of the CATALISE statements (Bishop et al., 2016, 2017), a few of the studies reviewed in this section used the term *DLD* instead of *SLI* (e.g., Adlof et al., 2020; Alt et al., 2019; Duff et al., 2020; Gray et al., 2019). For simplicity of presentation in this section, the term *SLI* is used throughout.

### Phonological Deficits

Deficits in the phonological domain of language have been featured in theories of both dyslexia (Lyon et al., 2003) and SLI (Gathercole & Baddeley, 1990; Leonard, 1989). Importantly, while children with persistent speech sound production deficits are more likely than those with typically developing (TD) articulation skills to develop SLI or dyslexia, most children with dyslexia or SLI have normal speech articulation skills (Cabbage et al., 2018; Shriberg et al., 1999). Thus, the phonological deficits discussed here generally involve the mental processing of phonological information, not the “phonological processes” typically associated with speech sound disorders.

A long-standing theory of dyslexia, the phonological core deficit model, posits that difficulties processing phonological information interfere with the ability to map phonemes to graphemes and vice versa (Lyon et al., 2003; Snowling, 1998; Vellutino et al., 2004). In dyslexia, the most significant phonological deficits involve phonemic awareness, the ability to reflect on and manipulate the speech sounds of language. Other phonological difficulties include the ability to retain strings of phonological information such as digits or nonsense words (i.e., phonological memory) and to rapidly retrieve and produce the names of visual symbols (i.e., rapid automatized naming).

In addition to problems with grammar, word learning, and semantic processing, children with SLI also frequently display difficulty in tasks involving phonological memory (Dollaghan & Campbell, 1998; Gathercole & Baddeley, 1990) and phonological awareness (Catts, 1993; Snowling et al., 2000). Gathercole and colleagues proposed that problems with the phonological loop of working memory could explain problems with language acquisition including word learning (Gathercole et al., 1999). A meta-analysis by Estes et al. (2007) demonstrated large group differences between children with SLI and TD children on nonword repetition tasks, underscoring its viability as a clinical marker of SLI.

Considering the frequent co-occurrence of language and reading difficulties, it was proposed that dyslexia and SLI might be different manifestations of the same underlying phonological deficit, distinguished by degree of severity (Kamhi & Catts, 1986; Tallal et al., 1997) or by the presence or absence of additional difficulties in other language domains (Bishop & Snowling, 2004). At the same time, it was possible that SLI and dyslexia did not actually share phonological deficits, but that previous finding of lower average performance relative to TD children in one of the disorders was explained by the presence of individuals with both disorders within the study sample. To date, numerous studies have examined phonological processing in SLI and dyslexia subgroups, with mixed results (Baird et al., 2011; Bishop et al., 2009; De Groot et al., 2015; Ehrhorn et al., 2020; Eisenmajer et al., 2005; Fraser et al., 2010; McArthur & Castles, 2013; Ramus et al., 2013; Rispens & Been, 2007; Robertson & Joannise, 2010). In their longitudinal study, Catts et al. (2005) found that phonological processing deficits, as measured by performance on phonological awareness and nonword repetition tasks, were more strongly associated with dyslexia than with SLI. However, findings from more recent studies have been less clear (see reviews in Adlof, 2017; Ehrhorn et al., 2020). Across studies and tasks, there is clear evidence that children with SLI + dyslexia show significant weakness across measures of phonological processing. However, when SLI-only and dyslexia-only (i.e., children with dyslexia who have average language skills) groups have been compared to TD children, some studies have found significant differences, whereas others have not. Similarly, mixed results have been found for comparing SLI-only to dyslexia-only. At present, it is unclear whether SLI-only and dyslexia-only can be distinguished on the basis of phonological processing abilities.

### Syntactic Processing

Syntactic deficits are part of the profile of SLI, but they have not been included as core features in the definitions of dyslexia. Yet, studies of children with dyslexia found that they sometimes showed difficulty relative to TD children on syntactic processing tasks (Mann et al., 1984; Rispens et al., 2004). This raised the question of whether SLI and dyslexia can be distinguished on the basis of syntactic processing abilities. Three studies compared children with dyslexia-only to children with SLI who showed a range of word reading abilities (including some in the range of dyslexia) on grammatical tasks assessing subject–verb agreement, past tense, relative clauses, and active and passive constructions (Rispens & Been, 2007; Robertson & Joannise, 2010; Robertson et al., 2013). Children with SLI performed significantly worse than children with dyslexia-only, but children with dyslexia-only exhibited subtle syntactic deficits relative to TD children, especially on tasks that taxed phonological memory resources. Another study by Cantiani et al. (2015) examined morphosyntactic processing in children with SLI + dyslexia and children with dyslexia-only with behavioral measures and event-related potentials. Results on behavioral measures of accuracy mirrored those of the previously cited studies, finding that children with SLI + dyslexia showed the poorest performance. Interestingly, the event-related potential results suggested qualitatively different processing between the dyslexia-only group and the SLI + dyslexia and TD groups. The authors concluded that children with dyslexia-only used qualitatively different processing to arrive at the same level of accuracy as TD children, whereas children with SLI + dyslexia used the same processing but were less efficient than TD children. Further research is needed to examine this hypothesis. Although stronger evidence would be provided by directly comparing children with SLI-only to those with dyslexia-only, the results of these studies converge to indicate that syntactic deficits are more closely associated with SLI than with dyslexia.

### Working Memory

Poor working memory has also been associated with both dyslexia and SLI, although the nature of the relations has been unclear (Alloway et al., 2009). Few studies had accounted for the comorbidity between SLI and dyslexia in the study of working memory, aside from those that examined phonological memory (reviewed in the Phonological Deficits section above). Addressing this gap in the literature, Gray et al. (2019) used latent class analysis to identify distinct working memory profiles within a large battery of tasks examining central executive, phonological, and visuospatial aspects of working memory and then examined whether each of the profiles was more or less associated with children in the dyslexia, SLI, SLI + dyslexia, or TD group. Participants were aged 7–9 years. Four profiles emerged from the working memory battery, with one profile representing overall high performance, one profile representing overall poor performance, and two intermediate profiles distinguished by higher or lower performance on a number updating task. Results indicated that the majority of children with SLI + dyslexia (66%) displayed the poorest overall working memory profile and that the majority of TD children (88%) displayed the highest overall working memory profile. The children in the SLI-only and dyslexia-only groups were somewhat more evenly distributed across all profiles. Overall, the results of Gray et al. did not support the notion of different working memory profiles for dyslexia versus SLI, as all working memory profiles were represented in all SLI and dyslexia subgroups as well as in TD children.

### Word Learning

Word learning difficulties have been repeatedly observed in children with SLI (Kan & Windsor, 2010) and dyslexia (Litt & Nation, 2014; Mayringer & Wimmer, 2000), but until recently, few studies had controlled for potential comorbidities, and no studies had directly contrasted the two disorders. Learning a new word is a complex task that draws on multiple language and cognitive skills and, therefore, may be differentially impacted in children with dyslexia and SLI. One recent study by Alt et al. (2019) contrasted the word learning abilities of second-grade students in three groups: dyslexia-only, SLI + dyslexia, and TD. Children learned pseudoword names and novel objects within paired-associate tasks that manipulated phonological and visual-semantic demands (e.g., naming and mispronunciation tasks that manipulated the form characteristics of words; visual feature recall tasks where the similarity of features was manipulated). Results showed that both groups of children with dyslexia (dyslexia-only and SLI + dyslexia) showed significantly poorer performance than TD children on most tasks that taxed phonological knowledge. There were fewer group differences overall on tasks that tapped visual-semantic skills; where differences were found, they were generally between the TD and SLI + dyslexia groups. Overall, the study showed that children with concomitant SLI + dyslexia showed the greatest difficulty learning new words.

Another study by Adlof et al. (2020) examined phonological versus semantic aspects of word learning in children from all four SLI and dyslexia subgroups (SLI-only, dyslexia-only, SLI + dyslexia, and TD) using a different methodology. Whereas the Alt et al. (2019) study used paired-associate tasks, this study explicitly taught a semantic description for each word–object pair. The instruction included multiple teaching exposures and incorporated spaced retrieval practice opportunities to facilitate word learning. Phonological versus semantic aspects of word learning were assessed immediately after instruction with tasks that required recall or recognition of the phonological and semantic information. Semantic recall was tested with verbal (elicited description) and nonverbal (drawing) tasks. Results indicated that, relative to TD children, children in the dyslexia-only and SLI + dyslexia groups showed broad deficits across all assessments of word learning. In contrast, children in the SLI-only group showed significant differences from children in the TD group on a single task that assessed verbal semantic recall. Although group differences were nonsignificant, the SLI-only group also scored higher than the dyslexia-only group, with moderate effect sizes on all tasks except the verbal semantic recall task. Taken together, these results suggested that children with dyslexia showed broader and more severe word learning deficits than children with SLI-only. The results were surprising, considering that, on norm-referenced measures of expressive and receptive vocabulary knowledge, children with dyslexia scored at or above the population mean, whereas children with SLI scored in the low-average range. Additional studies are needed to replicate these findings and examine factors to explain the differences in existing vocabulary size versus new word learning abilities for the dyslexia-only and SLI-only groups. Two possibilities include potential group differences in the ability to learn from naturalistic versus explicit and highly structured input as well as in the long-term retention of learned information. Examining these hypotheses may reveal important information to guide specialized treatments for these different disorders. Importantly, both Alt et al. and Adlof et al. (2020) found differences in word learning in children with single deficits versus SLI + dyslexia, underscoring the importance of considering comorbidities in future studies of dyslexia and/or SLI.

### Academic Progress

Although there is evidence that SLI and dyslexia both impact academic skills, including reading and math, relatively few studies have examined functional outcomes with measures used by school systems (e.g., Conti-Ramsden et al., 2009; Willcutt et al., 2007). One recent study is the first to account for the comorbidity of language impairment and dyslexia in examining school-administered measures of academic achievement (Duff et al., 2020). Duff et al. (2020) examined progress on school-administered, omnibus measures of reading and math achievement from second through fourth grades in a large sample (*N* = 448) of children who could be classified as having SLI-only, dyslexia-only, SLI + dyslexia or TD based on researcher-administered, norm-referenced measures of word reading and oral language. (Although Duff et al. did not implement a nonverbal IQ cutoff as an inclusionary criterion, over 92% of the children with language impairment scored within normal limits on the measure of nonverbal intelligence, suggesting that this sample would be largely similar to samples in past studies of SLI.) Reading and math achievement was measured twice yearly from second through fourth grades with a computerized adaptive assessment administered by the schools. At the first point of assessment, children with SLI + dyslexia scored lower than children with dyslexia-only and SLI-only, who, in turn, scored lower than TD children across all time points in both reading and math. Growth in reading and math was best explained with a quadratic function with faster growth in the earlier time points that tapered off over time, and all disordered groups showed persistently poorer performance than the TD group. Despite these significant and persistent differences in academic achievement, the majority of children who were classified into the dyslexia and/or SLI groups (> 60% across groups) had not received any type of special education services according to parent or school report, and children in the SLI-only group were least likely to have received services (7%–14% for SLI-only vs. 15%–33% for dyslexia-only and 31%–37% for SLI + dyslexia). These results highlight the functional importance of considering separate versus comorbid reading and language difficulties as well as a need to raise awareness of both disorders to improve identification and access to services.

### Summary

There is now a large body of research demonstrating that SLI and dyslexia are separate disorders: Children with SLI exhibit a range of word reading skills, and children with dyslexia exhibit a range of oral language abilities. However, the two disorders frequently co-occur. It is important to consider this co-occurrence in studies of each disorder. Otherwise, the results of studies of one disorder will be influenced by the presence of participants with the other disorder in the study sample.

Across the studies in this review, a common finding is that children with SLI + dyslexia showed the most severe deficits in each domain of study. Depending on the focus of study, children with SLI-only or dyslexia-only may perform more similarly to TD children or to children with SLI + dyslexia. To date, a majority of studies directly comparing these groups have examined phonological processing, with mixed findings overall. Beyond those studies examining phonological processing, very few studies have included children from all four groups (SLI-only, SLI + dyslexia, dyslexia-only, and TD). Thus, more studies are needed to clarify the language and cognitive profiles of SLI-only versus dyslexia-only groups and to better understand how comorbidity relates to severity of impairment. Based on the current data available, it is not possible to determine from an early age which children will develop SLI, dyslexia, or both (Snowling & Melby-Lervåg, 2016). Although it seems unlikely that a single core deficit can fully predict language and reading profiles (Astle & Fletcher-Watson, 2020; Catts et al., 2017; Pennington et al., 2012), studies that compare SLI-only to dyslexia-only can help determine which characteristics are most associated with either disorder or both disorders. Furthermore, it will be useful to consider protective factors, in addition to risk factors, associated with each disorder.

Clinically, it is clear that these groups experience significant and persistent functional impacts, as evidenced by school-administered measures of academic progress in reading and math. Although SLI-only and dyslexia-only groups in the Duff et al. (2020) study scored similarly on omnibus measures of reading achievement, they will need different kinds of reading intervention to address their different sources of reading difficulties. The same could be true for math (see Moll et al., 2015), but more research is needed. Finally, the large percentage of students in all groups, especially the SLI-only group, who had not previously been identified as in need of educational supports highlights a need to raise awareness of these disorders and their impacts as well as for further research into effective practices for identification and service delivery.

## Identification of Children With SLI or Dyslexia

Evidence suggests that both SLI and dyslexia are underdiagnosed (Adlof et al., 2017; Phillips & Odegard, 2017; Tomblin et al., 1997). For example, in past studies employing population- or community-based samples, less than 40% of children with language impairment had been identified for clinical or educational services (Adlof et al., 2017; Norbury et al., 2016; Tomblin et al., 1997). Rates of underidentification also differ according to demographic factors, such that children from minority racial, ethnic, and linguistic backgrounds; from households with lower socioeconomic status; or with mothers who have lower education levels are less likely to receive special education services than White and middle-class peers (P. L. Morgan et al., 2017, 2016; Wittke & Spaulding, 2018). However, some evidence suggests that identification rates may be particularly low for children with SLI-only as compared to those with co-occurring dyslexia (Adlof et al., 2017; Catts et al., 2005). Currently, most public schools rely on different assessment frameworks to identify children with speech and oral language difficulties versus reading difficulties.

### Current Identification of Language Impairment

In the United States, most schools rely on a referral process for the identification of children with speech and language impairments. When a parent, a teacher, or other caregiver has reason for concern about a child's language development, an evaluation from a speech-language pathologist (SLP) may be requested. However, there are several challenges with referrals that likely play into low rates of identification of SLI. First, there appears to be a lack of public awareness of language disorders in general (Bishop et al., 2012; Schuele & Hadley, 1999). Terminology around both dyslexia and SLI has evolved over time (Elliot & Grigorenko, 2014; Leonard, 2020), but evidence points to greater public awareness of dyslexia than of SLI (Bishop, 2010). In this regard, efforts to raise awareness of language disorders overall may be helpful to improve identification.

A lack of awareness may be compounded by the fact that the signs of language impairment are relatively difficult to track without direct measurement. Whereas most parents and teachers can recognize when children have difficulty producing speech sounds, the signs of language impairment are more subtle. Importantly, speech impairment and language impairment are independent in 6-year-old children (Shriberg et al., 1999). However, school-based SLP caseloads contain more children with combined speech articulation disorders and language impairment even though children with SLI who have normal speech articulation skills are far more prevalent in the population (Zhang & Tomblin, 2000). Children with SLI make more grammatical errors than their peers, but most of what they say is grammatically accurate (Rice et al., 2004). Conversational language tends to be relatively simple in relation to what a TD child is able to comprehend or produce (Nippold, 2009), which may obscure individual differences in higher level language skills. Thus, breakdowns in communication may not be perceived as such and could instead be attributed to a variety of factors, including shyness, inattention, forgetfulness, or lack of motivation. For these reasons, language impairments have been described as “hidden” (Bishop, 2014; Nation et al., 2004). Indeed, Adlof et al. (2017) and Hendricks et al. (2019) found that parents of children who were found to have significant language weaknesses rarely indicated concerns about their children's language abilities in response to targeted questions on study intake questionnaires. Other evidence suggests that classroom teachers also have difficulty discriminating normal from disordered language abilities (Christopulos & Kean, 2020; Jessup et al., 2008; C. Williams, 2006). Taken together, a lack of awareness of language impairment overall and a lack of sensitivity to children's potential language difficulties may contribute to underreferral for evaluation.

Second, the diagnostic decision process for SLPs is usually framed in a binary way: impaired versus not impaired. In general, only children who are diagnosed as impaired receive intervention. As there can be harmful effects of misdiagnosis, much emphasis has been placed on having accurate diagnostic tests to inform these binary decisions (Spaulding et al., 2006). Valid and reliable tests are available to detect language impairment in monolingual English speakers beginning in the preschool years (Spaulding et al., 2006). Unfortunately, resources for the accurate diagnosis of language ability in children who speak more than one language and in those who speak a dialect that differs from the mainstream are more limited (Muñoz et al., 2014; but see Peña et al., 2018; Seymour et al., 2005). Additionally, concerns about diagnosing language differences as language disorders, combined with the use of nonvalidated assessment practices, may contribute to higher levels of under-identification of language impairment for these children, limiting their access to instructional supports to promote academic progress (Hendricks & Adlof, 2017; Hendricks & Diehm, 2020; Oetting et al., 2016; Samson & Lesaux, 2009).

Another limitation associated with current binary diagnostic decisions is that many children with subclinical spoken-language weaknesses—who score just above the cutoff for language impairment—exhibit more serious difficulties with reading comprehension where the linguistic demands are more challenging (Adlof & Catts, 2015; Catts et al., 2006; Nation et al., 2004). Because of the developmental shift in the contribution of decoding versus listening comprehension skills on reading comprehension, the impact of language impairment on academic performance may remain hidden until the middle elementary grades or later (Catts et al., 2012; Lipka et al., 2006). Nonetheless, direct measurement of spoken language can reveal oral language weaknesses that are present prior to the initiation of formal reading instruction (Fong & Ho, 2019; Justice et al., 2013; Nation et al., 2010). For example, Catts et al. (2012) found that 70% of children with SLI in kindergarten exhibited a reading impairment at some point between second and 10th grades. However, about 40% of those were “late emerging,” meaning that reading test scores fell within normal limits until the fourth-grade assessment or later. Justice et al. (2013) demonstrated that fifth-grade poor comprehenders (who showed poor reading comprehension in spite of strong word reading abilities) showed significantly poorer oral language skills from 15 months of age; further analyses showed that their level of spoken language, relative to same-age peers, declined between 15 and 54 months of age (Petscher et al., 2018). Such findings suggest the importance of monitoring the development of oral language skills to support reading comprehension for all students.

### Current Identification of Word Reading Impairment

Until formal reading instruction has been provided, dyslexia is just as “hidden” as SLI. Among scientists who study dyslexia, it is commonly recognized that dyslexia represents the lower end of a continuous distribution of word reading ability (Boada et al., 2012; Miciak & Fletcher, 2020). However, distinguishing differences in a child's innate ability from differences related to the quality and quantity of reading instruction a child has received can be quite difficult. Some popular reading curricula do not feature explicit instruction and lack scientific evidence of effectiveness. Additionally, while risk factors for dyslexia can be measured in the preschool years, the presence of risk factors does not guarantee a reading difficulty (Catts et al., 2017; Snowling & Melby-Lervåg, 2016). Furthermore, measurement issues make it difficult to accurately assess word reading skills before children have had a sufficient amount (e.g., 1–2 years) of formal reading instruction. Because most children enter school unable to read many words, measures of word reading—as well as foundational skills such as phonological awareness and letter-sound knowledge—may show floor effects in kindergarten (Catts et al., 2009). As children progress through formal reading instruction, greater variation between students can be observed, allowing for better identification of children with very low performance. Unfortunately, waiting for this variation to emerge before providing intervention means missing a critical window for providing early intervention. Reading outcomes are significantly better when reading difficulties are treated earlier (Al Otaiba et al., 2014; Lovett et al., 2017).

In light of these issues, response to intervention (RTI) frameworks were introduced to both identify children in need of supportive services and provide necessary supports as quickly as possible, and the 2004 reauthorization of the Individuals with Disabilities Education Act (Public Law 108-446) allowed RTI to be used for determining eligibility for special education services. RTI frameworks are prevention oriented and integrate assessment and intervention across multiple tiers. It is expected that students in all tiers receive high-quality, evidence-based instruction. Within the first tier, all students participate in universal screens to identify risk for reading difficulties, and their reading progress is monitored at regular intervals (e.g., quarterly). Students who are flagged as “at-risk” by the screens and those who fail to make adequate progress in response to evidence-based instruction are moved into increasingly higher tiers, where the instruction is more explicit and more intensive (e.g., in smaller groups, with more instructional time) and where progress is monitored more frequently (e.g., weekly or monthly). Students who demonstrate a need for the highest levels of support may be considered for special education services. Although RTI frameworks can be applied across grades, universal screens and progress monitoring for word reading skills are most commonly recommended for students in kindergarten through second grade (e.g., Gersten et al., 2008).

In the ideal implementation of RTI, movement through the tiers is fluid, efficient, and data driven, and the risk of long-term reading disability is minimized because intervention is provided swiftly and differentiated according to students' level of progress and need. The value of RTI when implemented well is that it is a whole-school framework (i.e., it serves *all* students, not just those with identified impairments), and it is prevention oriented, aiming to provide intervention and close gaps as early as possible to increase the likelihood of positive long-term outcomes. Because of its focus on progress toward academic outcomes of interest, RTI can be helpful for culturally and linguistically diverse populations who are at risk of both under- and overidentification within traditional identification frameworks that rely on referrals and binary diagnostic decisions. In general, RTI is not concerned with diagnostic labels but is rather focused on calibrating the level of instructional support to meet the child's needs so that they make adequate educational progress. This means that children with dyslexia may not be identified as such if they are making appropriate educational progress within their designated RTI tier. However, it is important to note that RTI cannot be used to delay or deny an evaluation for special education eligibility (Musgrove, 2011); rather, the purposes of RTI are to facilitate earlier identification of risk and earlier access to targeted interventions and to promote academic progress. Multiple studies have shown the promise of RTI frameworks for improving reading outcomes for at-risk students when interventions are implemented consistently, at the appropriate intensity, and with fidelity (Coyne et al., 2018; Wanzek et al., 2016).

### Potential for Including Oral Language Assessment in RTI Frameworks

Most schools in the United States currently use some form of RTI for reading in the elementary grades, although the quality of implementation is variable (Balu et al., 2015; Coyne et al., 2018; Fuchs & Fuchs, 2017). Concerted advocacy efforts to raise awareness of dyslexia have led to the passage of dyslexia legislation in 46 of the 50 states (Eide, 2020; Ward-Lonergan & Duthie, 2018). These laws are intended to provide more specific guidance regarding the characteristics and needs of children with dyslexia, and many states have mandated universal screening for dyslexia in kindergarten through second grade. However, aside from phonological awareness and vocabulary knowledge, oral language is not generally addressed within these mandates. RTI has been discussed with regard to school-based SLP practices for some time (Ebbels et al., 2019; Ehren & Whitmire, 2009; Sanger et al., 2012), but research and resources are needed to support full implementation. An important first step is the addition of direct measurement of oral language skills to existing RTI frameworks, including both universal screens and progress monitoring assessments. Incorporating these measurements will allow for increased identification of children with SLI and those with low-average language skills who are likely to struggle with reading comprehension. Earlier identification of risk, in turn, allows for earlier access to interventions to promote positive outcomes.

#### Universal Screening

With universal screening, all students in a class or school are briefly assessed to identify students who may be at risk for poor learning outcomes and require closer monitoring. It is important that screens have both high sensitivity (accurate classification of children with language impairment) and high specificity (accurate identification of children with typical language abilities) because errors mean that diagnostic and/or intervention resources will be allocated to the wrong students. Additionally, screens that require minimal training and can be administered and scored efficiently are generally preferred for universal administration.

Currently, a number of published oral language screens for school-aged children are available, but psychometric information to support their use as universal screens is somewhat limited. Some screening manuals report high levels of sensitivity and specificity (e.g., Nelson et al., 2018; Wiig et al., 2013) based on pre-identified clinical (or risk) and TD samples. It remains to be determined whether sensitivity and specificity remain adequate in universal screening applications. A few recent studies have evaluated researcher-administered protocols that suggest the promise of universal screening for language. For example, Redmond et al. (2019) found that individually administered screens involving sentence repetition and past-tense elicitation tasks have high sensitivity and specificity against a range of reference standards for language impairment (see also Archibald & Joanisse, 2009). Whereas Redmond et al.'s study focused on monolingual English speakers, Pratt et al. (2020) demonstrated the utility of sentence repetition tasks as a screen for language impairment in bilingual Spanish–English students (see also Gutiérrez-Clellen et al., 2006). Adlof et al. (2017) and Hendricks et al. (2019) have demonstrated the potential of group-administered screenings using sentence–picture matching tasks for identifying language impairment within samples that included speakers of mainstream and nonmainstream dialects of American English, although the sensitivity and specificity for the group-administered tasks were lower than those achieved with individually administered measures in the work of Redmond et al. In some situations, the benefits in terms of screening efficiency may outweigh the costs of reduced classification accuracy of group screening instruments. However, further development of group screening instruments may yield tools with similar levels of classification accuracy as individually administered measures. Computerized methods may also be useful for efficient universal screening by reducing the requirement for trained examiners or scorers. For example, the Lexia RAPID Assessment (n.d.) includes measures of “academic language” that are known to influence reading comprehension. Additionally, Grammaggio (Rice, n.d.) is a free mobile app that measures children's ability to make grammaticality judgments involving structures that are known to be difficult for children with SLI who speak mainstream American English. Taken together, there are multiple approaches that show promise for further research and development as universal screens for language within an RTI framework.

#### Progress Monitoring

Whereas a screening instrument is used to assess risk based on a single point in time, progress monitoring provides information about the rate of change in a particular skill and helps determine when an instructional change is needed. Progress monitoring assessments are repeated at regular intervals, with higher frequency for students who are receiving instructional supports in Tier 2 or Tier 3, rather than Tier 1, of an RTI framework. If a student's rate of growth is not sufficient to meet their learning goals, the student may need a different or more intensive instruction. When implemented correctly, progress monitoring can improve access to instructional supports for children who fall outside the screening cutoff yet struggle to make progress or for whom the validity of a particular screening instance may be in question (e.g., students from culturally and linguistically diverse backgrounds).

As with any assessment, a progress monitoring instrument needs to be valid and reliable. In addition, progress monitoring requires multiple equated test forms or tasks that are sensitive to change over time. For example, to measure progress in word reading for students in Tier 2 intervention, a teacher might take a weekly measure of the number of words read correctly per minute on grade level passages. Currently, there are limited resources for monitoring progress in the language skills that support reading comprehension across different academic content areas, that is, vocabulary, syntax, and discourse. For children in kindergarten through third grade, the Narrative Language Measures (Petersen & Spencer, 2012) includes multiple brief stories of the same length, vocabulary level, and grammatical complexity, which were specifically developed to monitor progress in narrative discourse skills. Language samples may be useful for measuring progress in complex syntax and discourse, but it is important to consider the type of task (expository, narrative, or conversation; written vs. oral) used to elicit the language sample (Nippold et al., 2005, 2009). Progress monitoring in vocabulary is challenging, due in part to the huge set of possible words that could be assessed and the differing ways to define “knowing” a word. Some researchers have used elicited definitions (Duff, 2019; Storkel et al., 2019) to evaluate the depth of word knowledge on a graded scale. As a complement to definition generation tasks, Duff (2019) described the use of Context Test Questions, which measure the ability to recognize correct usage of a word across contexts, from very general to very specific.

Overall, there are multiple research-based tasks that can be used to guide the development of progress monitoring tools, but further evidence of reliability and validity will be needed. Additionally, most of these discussed here are relatively labor intensive and require a skilled assessor. Implementation of measures such as these in an RTI framework may be challenging considering the workload of current school-based SLPs. Thus, more research is needed to develop feasible, valid, and reliable procedures for measuring growth across the different language domains that support reading comprehension and academic communication.

## Conclusions

Successful reading comprehension relies on two sets of component skills: word reading and language comprehension. Dyslexia and SLI each contribute risk to reading comprehension through their negative influence on one of the components, that is, word reading for dyslexia and language comprehension for SLI. There is now extensive evidence that dyslexia and SLI are different disorders that frequently co-occur. However, the two disorders have largely been studied separately. Research that considers separate versus co-occurring cases is needed to advance the understanding of the characteristics and potential causes of each disorder. However, underidentification, especially for children with SLI who have adequate word reading abilities, presents a challenge for such research. Additionally, most previous studies have focused on monolingual English speakers, and there is a need for more research involving students from diverse language backgrounds.

Existing school RTI frameworks are heavily weighted toward word reading skills, with little attention to the direct monitoring of language abilities. A dependence on referral models for language disorders, combined with the “hidden” nature of language problems, may contribute to the under-identification of SLI. Thus, SLPs have an important role to play in raising awareness of the direct role of broader oral language skills, including vocabulary, syntax, and discourse, in reading comprehension. Considering the frequent comorbidity of dyslexia and SLI, all school-aged children who are identified with word reading problems should receive a thorough language evaluation. Additionally, there is potential for much to be gained by directly monitoring the language skills of all students within an RTI framework, including improved identification of children with SLI as well as access to interventions and compensatory resources for children with SLI and those with subclinical language weaknesses. Research is needed to further the development of measurement tools that are reliable, valid, and feasible for use with students from diverse cultural and linguistic backgrounds.

## Footnote

1
*SLI* and *DLD* are conceptually similar terms, but it is often understood that DLD accepts a wider range of nonverbal IQ scores than SLI (e.g., Leonard, 2020; Norbury et al., 2016). Although studies of SLI have frequently required nonverbal IQ standard scores of 85 or higher, some studies have accepted scores as low as 70 (Gallinat & Spaulding, 2014). This clinical focus article uses the term *SLI* in keeping with the 2019 American Speech-Language-Hearing Association Research Symposium title.
